# Islet-1 May Function as an Assistant Factor for Histone Acetylation and Regulation of Cardiac Development-Related Transcription Factor Mef2c Expression 

**DOI:** 10.1371/journal.pone.0077690

**Published:** 2013-10-17

**Authors:** Zhongsu Yu, Juanjuan Kong, Bo Pan, Huichao Sun, Tiewei Lv, Jing Zhu, Guoying Huang, Jie Tian

**Affiliations:** 1 Heart Centre, Children's Hospital of Chongqing Medical University, Chongqing, PR China; 2 Key Laboratory of Developmental Disease in Childhood (Chongqing Medical University), Ministry of Education, Chongqing, PR China; 3 Pediatric Heart Centre, Children's Hospital of Fudan University, Shanghai, PR China; Baylor College of Medicine, United States of America

## Abstract

**Objective:**

Islet-1 is an important transcription factor for cardiac development through mediating extensive interactions between DNA and proteins. The present study was to investigate the role of Islet-1 in regulating the expression of cardiac development-related transcription factors and mechanism.

**Methods and Results:**

The expression of Islet-1 and histone acetylases (HATs) subtype p300 was determined in newborn mouse hearts and mouse embryonic hearts at different development stages using Western blot. The expression of Islet-1 and cardiac development-related transcription factors Mef2c, GATA4 and Tbx5 as well as histone H3 acetylation level were determined in cardiac progenitor cells with and without transfection of Islet-1 interference RNA (RNAi) in lentivirus using PCR and Western blot. Islet-1 peak expression occurred on day E14.5 in mouse embryonic heart, and was present in the promoter regions of Mef2c, GATA4 and Tbx5 that were precipitated with p300 antibody. When Islet-1 was inhibited with specific RNAi in cardiac progenitor cells, the expression of Mef2c and Tbx5, but not GATA4, was significantly suppressed along with selective reduction in histone H3 acetylation in the promoter region of Mef2c, but not GATA4 and Tbx5. The level of Mef2c DNA, not GATA4 and Tbx5, in the complex associated with p300 was significantly decreased in the cells with Islet-1 knockdown.

**Conclusions:**

These data suggested that Islet-1 might function as an assistant factor that was involved in the regulation of histone acetylation and Mef2c expression via assisting p300 on specifically targeting the promoter of Mef2c.

## Introduction

Cardiac development is a complex process. Many transcripiton factors, such as Mef2c, GATA4, Tbx5, and Nkx2.5, involve in cardiac development, and are expressed in a sequential and stage-specific pattern in normal situation [1]. However, the expression profile of cardiac development-related transcription factors does not depend on DNA sequence. Instead, accumulating data suggest that the expression of these transcription factors is largely regulated through epigenetic, a mechanism that involves modifications of DNA or chromatin without changes in DNA sequence [2]. 

Major epigenetic regulations include: DNA methylation, histone modification, and microRNA ([3]; [4] [5];). Modification of histone acetylation is an important mechanism in the regulation of gene expression [6]. Histone acetylation is a reversible and dynamic process that is mediated via histone acetyltransferases (HATs) and histone deacetylase (HDACs) [7]. Our previous studies showed that several subtypes of HATs were dynamically expressed in embryonic heart with p300 as the most dominant one [8]. Inhibition of p300 would attenuate the expression of cardiac development-related transcription factors in vitro [9]. On the other hand, an increased expression of cardiac transcription factors was observed when histone hyperacetylation was induced with alcohol [10]. These observations indicated that histone acetylation was involved in regulating the expression of cardiac development-related transcription factors. However, it is unclear how histone acetylation regulates these transcription factors at the key time points during cardiac development. It is possible that HATs may need an assistant factor to recognize the cardiac development-related transcription factors. 

Islet-1 is a member of LIM-Homeodomain transcription factors that plays a pivotal role in cardiac progenitor cells in the second heart field [11]. These progenitor cells from the second heart lineage become part of the developing heart tube, and give rise to both the outflow tracts, the right ventricle, and the main part of the atrial tissue [12,13]. The hearts in the mice lacking Islet-1 are completely lack of the outflow tract, right ventricle and much of the atria [14]. Inhibition of Islet-1 interrupts normal looping of the linear heart tube, and results in a lethal heart deformity [15].. It is believed that there is an Islet-1-dependent transcriptional network for second heart field development, in which Islet-1 as a key early factor directly regulates cardiac development-related factor Mef2c expression (Dodou E et al., 2004). The LIM domains of Islet-1 mediate the interaction between proteins and homeodomain which in turn mediates the interaction between DNA and proteins[16]. Considering the function and structure of Islet-1, we speculated that Islet-1 might be an assistant factor that could regulate the expression of cardiac development-related transcription factors through mediating histone acetylation. 

## Materials and Methods

### Cardiac tissue preparation from mouse embryos

Healthy adult Kunming (KM) mice (weight 27-31g) were purchased from the Experimental Animal Center in Chongqing Medical University (Chongqing, China). All animal procedures were approved by the Animal Care and Use Committee at the Chongqing Medical University. Female mice were mated with the male partners. When a vaginal plug was found at noon of the following day, it was considered as embryo day 0.5 (E0.5). On E11.5, E14.5, and E17.5, the pregnant mice were sacrificed through cervical dislocation, and the embryonic hearts were collected. The hearts of 1-day-old neonatal mice were also collected. 

### Culture of cardiac progenitor cells and vector transfection

Cardiac progenitor cells, as a kind gift from the Molecular Oncology Laboratory at the University of Chicago Medical Center [17], were cultured in Dulbecco’s modified Eagle medium (DMEM) (Thermo, Waltham, MA, USA) containing 10% fetal bovine serum (FBS) (Hyclone, Logan, USA) in humidified air with 5% CO_2_ at 37°C. When the cells reached 90% confluence, they were subculture for 2 passages. The cells at the third passage were used for our experiments. The cells were randomly divided into three groups: Islet-1 RNAi group (the cells were transfected with lentivrius with Islet-1 RNA interference vector), negative control group (the cells were transfected with control lentiviurs), and blank control group (the cells were not transfected with any vector). The lentivrius with Islet-1 RNA interference vector and the control lentivirus were prepared and introduced into the cells as previously described in detail [18]. Both of the vectors expressed green fluorescent protein (GFP). The transfection efficiency was quantitatively determined using flow cytometry. 

### Western blotting analysis

Nuclear proteins were extracted from the hearts and cardiac progenitor cells with different treatment using nuclear extract kit (Keygen, Nanjing, China) according to the manufacturer’s instructions. The proteins were separated via electrophoresis on 15% Bis-Tris polyacrylamide gels, and then transferred to polyvinylidene difluoride (PVDF) membrane (Millipore, MA, USA). After incubating in 5% nonfat milk for 1 hr, the preparations were exposed to rabbit polyclonal antibody against Islet-1 (Millipore, MA, USA, 1:200 dilution) or rabbit monoclonal antibody against acetylated histone H3 (acH3) (Millipore, MA, USA, 1:1000 dilution), or mouse monoclonal antibody against β-actin (Abcam, Cambridge, Britain, 1:1000 dilution) in phosphate buffer solution (PBS) plus 5% nonfat milk and 0.05% Tween-20 at 4 °C overnight. After washing with PBS, the membranes were then incubated in HRP conjugated goat secondary antibody (anti-rabbit and anti-mouse) (Zhong Shan Golden Bridge, Beijing, China) with a dilution factor of 1:2000 for 1 hr at room temperature. Protein bands were revealed with an Enhanced Chemiluminescence Luminal reagent (Keygen, Nanjing, China), scanned and analyzed with Quantity One Version 4.4 software (Bio-Rad, CA, USA).

### Co-immunoprecipitation for p300 interaction

Nuclear proteins were extracted from the hearts using nuclear extract kit (Keygen, Nanjing, China) as per the manufacturer’s instructions. Immunoprecipitations were performed using normal IgG (for preclear), anti-p300 antibody (Abcam, USA), and protein A/G-agarose (Santa Cruz, CA) at 4°C. The lysates and immunoprecipitates were detected with Western blotting using Islet-1 primary antibody and appropriate secondary antibody, followed by quantitative analysis with SuperSignal chemiluminescence kit (Pierce).

### Chromatin immunoprecipitation (ChIP) assay

ChIP analysis was used to evaluate the level of histone H3 acetylation in the promoters of cardiac development-related transcription factors and to detect the interaction between these transcription factors and p300, as well as Islet-1 level. The cardiac progenitor cells with different treatment and embryonic heart tissue were collected and prepared for ChIP assay following the manufacturer's protocol using a ChIP assay kit (Millipore, MA, USA). Proteins and DNA were crossed link after adding formaldehyde into the cell or heart tissue samples. The DNAs were ultrasonically cut into small fragments. Then the protein-DNA complexes were recruited and precipitated using monoclonal antibody against Islet-1 (ChIP grade, Santa cruz, CA, USA), monoclonal antibody against acH3 or p300 (ChIP grade, Millipore, MA, USA). The protein-DNA complexes were also precipitated using anti-RNA polymerase II antibody as positive control and using normal mouse IgG as negative control. The protein-DNA complexes were also collected without antibody as input group (to show the total DNA in the samples). After removing the crossing link of proteins and DNAs, the DNAs were extracted. Specific primers were designed to determine the acetylation level of hitone H3 in promoter regions of cardiac development-related transcription factors and the interaction of p300 or Islet-1with these transcription factors for quantitative Real-Time PCR (Q-PCR) analysis. The primers’ sequences were as follows: Mef2c: 5’-CACGCATCTCACCGCTTGACG-3’ (upper), and 5’-CACCAGTGCCTTTCTGCTTCT- CC-3’ (lower); GATA4: 5’-CACTGACGCCGACTCCAAACTAA-3’ (upper), and 5’-CGACTGGGGTCCAATCAAAAGG-3’ (lower); Tbx5: 5’-TCTAAGCCGTTCTGGAGC- CCGACA-3’ (upper), and 5’-AGAGCCTCCCAGCGACTGCCCAC-3’ (lower). Q-PCR assay was performed using SYBR Green RealMasterMix kit (Tiangen, Beijing, China) as per manufacturer’s instructions. The annealing temperature was 68 °C for Mef2c and Tbx5, 60°C for GATA4. Products of Q-PCR were measured using polyacrylamide gel electrophoresis.

### Quantitative RT-PCR analysis

Total RNAs were extracted from the cardiac progenitor cells of three groups using RNA extract kit (Bioteck, Beijing, China). First-strand cDNA was synthesized from 500 to 1000 ng RNA by using oligo dT-Adaptor primers and AMV reverse transcriptase (TaKaRa, Otsu, Japan) according to the manufacturer's instructions. The cDNA was detected using quantitative RT-PCR assay with SYBR Green RealMasterMix kit (Tiangen, Beijing, China) to determine the transcriptional expression of cardiac development-related transcription factors including Mef2c, GATA4, and Tbx5. β-actin was used as the endogenous “house-keeping” gene to normalize the RNA sample levels. The primers’ sequences were as follows: Islet-1: 5’-ACACCTTGGGCGGACCTGCTATG-3’ (upper), and 5’-TGAAACCAC- ACTCGGATGACTCTG-3’ (lower); Mef2c: 5’-GCGCAGGGAATGGATACGG-3’ (upper) and 5’-TGCCAGGTGGGATAAGAACG-3’ (lower); GATA4: 5’-CCCTCCCGCACGATTT- CT-3’ (upper), and 5’-AGAGGCCCAACTCGCTCAA-3’; Tbx5: 5’-CCAAAGACAGGTCT- TGCGATTCG-3’ (upper), and 5’-TTCTCCTCCCTGCCTTGGTGAT-3’ (lower); β-actin: 5’-CACACCCGCCACCAGTTCG-3’ (upper), and 5’-GTCCTTCTGACCCATTCCCACC-3’ (lower). The annealing temperature was 63.7°C for Islet-1, and 60°C for Mef2c, GATA4, Tbx5, and β-actin. The relative mRNA expression was determined using 2^-△△Ct^ method.

### Statistical analysis

All data were reported as mean±SD, and statistically analyzed using one-way ANOVA. The difference was considered to be statistically significant when a P value <0.05.

## Results

### Islet-1 protein was dynamically expressed in normal embryo hearts

**Figure 1:**
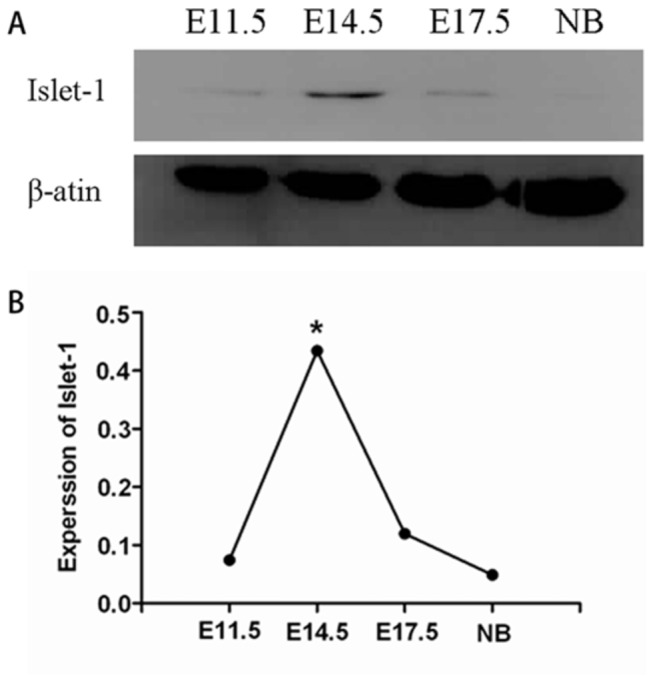
Expression of Islet-1 protein in mouse embryonic and neonatal hearts. A representative Western blotting showed that Islet-1 was dynamically expressed in the embryonic hearts. The expression of Islet-1 in mouse embryonic hearts was significantly higher on E14.5 than that on E11.5, E17.5, and neonatal mice. * *p* < 0.05 (n=3).

Western blotting analysis demonstrated that Islet-1 was dynamically expressed in embryo hearts during development. Detectable level of Islet-1 was present in the heart on day E11.5 (0.074±0.023). It reached the peak level on day E14.5 (0.434±0.176), and then was gradually decreased afterwards (Figure **1A** and Figure **1B**) (*p*<0.05). Interestingly, no detectable level of Islet-1 was observed in the neonatal heart. Therefore, the cardiac tissues for the embryos on day E14.5 were used for the present study. 

### Islet-1 and p300 were present in the same protein complex in embryonic heart

**Figure 2:**
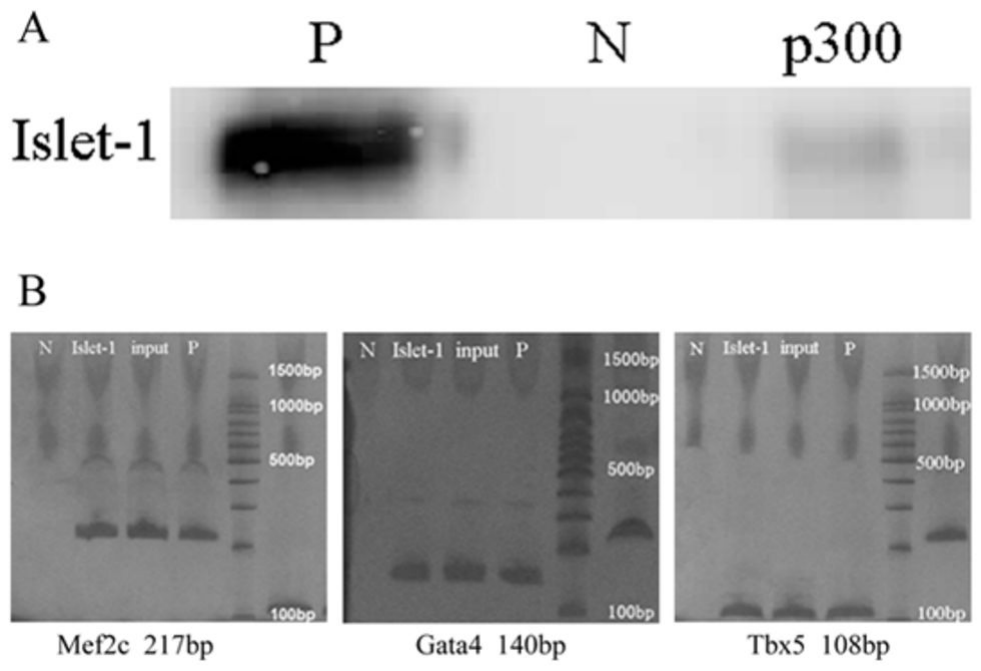
Islet-1 protein interacted with p300 cardiac development-related transcription factors in embryonic mouse heart. Islet-1 protein interacted with p300 in embryonic mouse heart (A). After precipitation using CoIP, Western blotting showed that Islet-1 protein was present in the protein complex precipitated with p300 antibody in p300 group, and positive control group, not in the negative control group. p300: protein complex precipitated with anti-p300 antibody; P: positive control without CoIP; N: negative control, protein complex precipitated by anti-IgG antibody. Islet-1 protein interacted with cardiac development-related transcription factors in embryonic mouse heart (B). After amplifying the DNA fragments from ChIP using Q-PCR, the polyacrylamide polyacrylamide gel electrophoresis data demonstrated that cardiac development-related transcription factors Mef2c, GATA4, and Tbx5 were present in the preparations precipitated with anti-Islet-1 antibody, anti-RNA polymerase II antibody (for positive control), and without antibody (input control), but not in negative control group. Islet-1: amplified with DNA precipitated by anti-Islet-1 antibody; input: amplified with DNA without antibody precipitation; P: positive control, amplified with DNA precipitated by anti-RNA polymerase II antibody; N: negative control, amplified with DNA precipitated by normal mouse IgG.

Using CoIP and Western blotting, we found that Islet-1 protein was present in the protein complex in cardiac tissues isolated from the embryo mouse hearts on E14.5. Islet-1 protein was also found in the protein complex precipitated with p300 antibody as shown in Figure **2A**. As expected, no Islet-1 protein was identified in negative control (using anti-IgG antibody to precipitate protein complex) (Figure **2A**). These data suggested that Islet-1 could interact with p300 in cardiac tissues from mouse embryonic hearts.

### Islet-1 was present in the promoter regions of cardiac development-related transcription factors

DNA fragments from the preparations precipitated with Islet-1 antibody were amplified by Q-PCR using specific primers for the promoters of cardiac development-related transcription factors. The Q-PCR products were analyzed with polyacrylamide gel electrophoresis, and showed that a significant amount of PCR products for the promoters of Mef2c, GATA4, and Tbx5 were present in the preparations precipitated with anti-Islet-1 antibody, anti-RNA polymerase II antibody (for positive control), and without antibody (input control). No detectable PCR products for these promoters were present in the negative control preparations (using normal mouse IgG) as expected (Figure **2B**). These results suggested that Islet-1 protein was present in the promoter regions of Mef2c, GATA4, and Tbx5, might interact directly or indirectly with these transcription factors in the mouse embryonic hearts.

### Inhibition of Islet-1 significantly suppressed the expression of cardiac development-related transcription factors in cardiac progenitor cells

**Figure 3:**
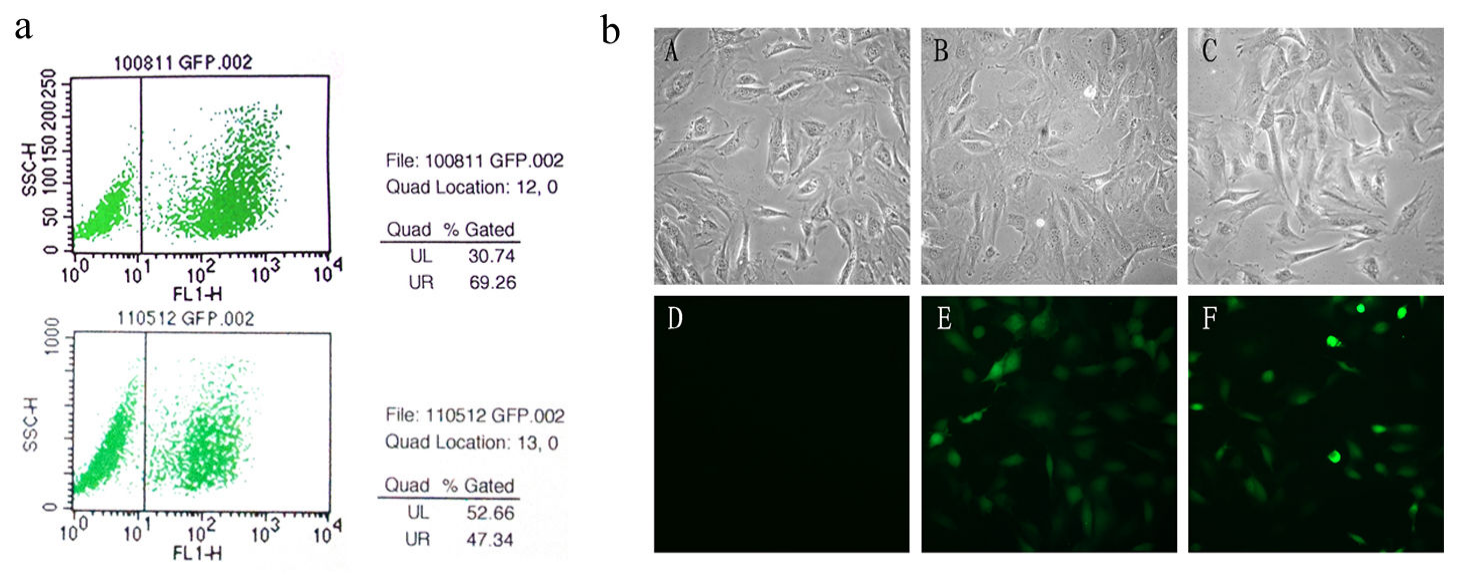
Flow cytometry analysis and the morphology of cardiac progenitor cells (×200). Flow cytometry analysis showed that the transfection rate was 69.26% in the cells transfected with control vectors (A) and 47.34% in the cells transfected with Islet-1 RNAi vector (B) (a). The morphology of cardiac progenitor cells (×200) (b) did not change after vector transfection. A: normal control; B: empty vector control; C: Islet-1 RNAi group; D. GFP expression in blank control; E. GFP expression in the empty vector control; F. GFP expression in the cells transfected with Islet-1 RNAi vector.

**Figure 4:**
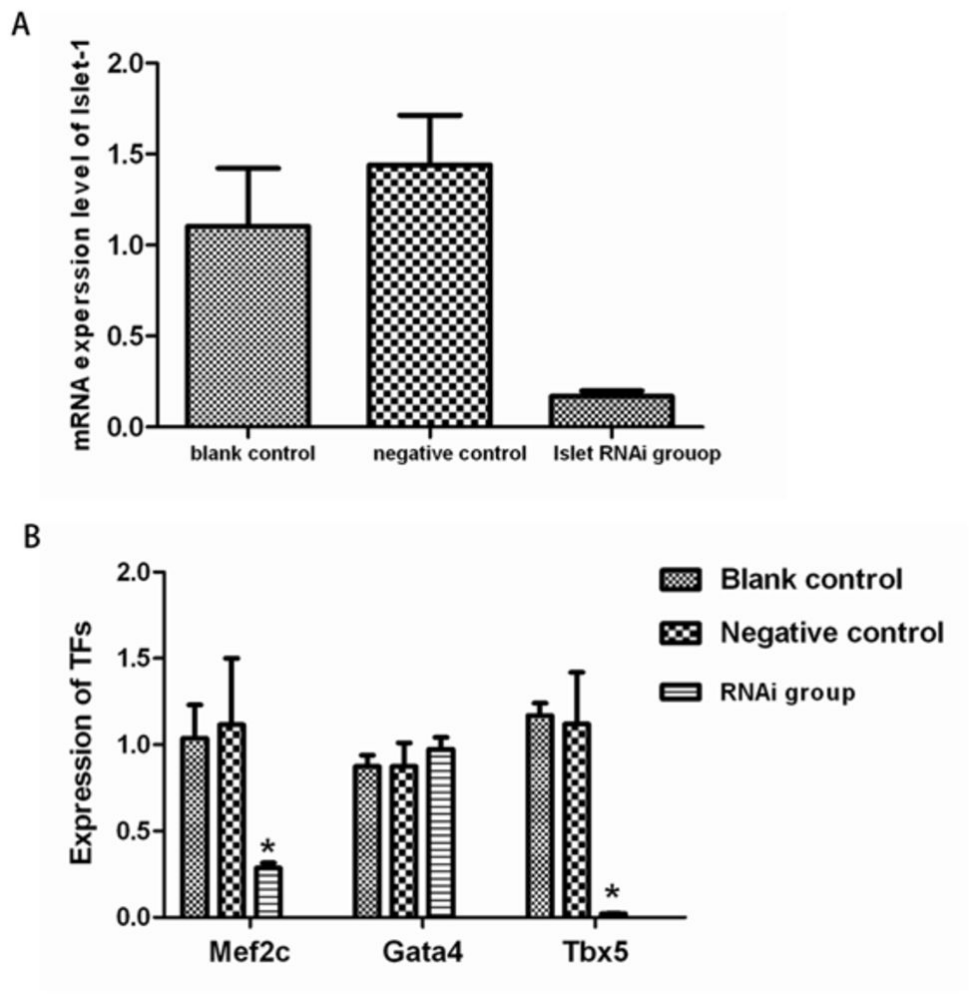
Transcriptional expression of cardiac development-related transcription factors in cardiac progenitor cells after lentivirus transfeciton with Islet-1 RNAi. Quantitative RT-PCR analysis showed the mRNA level for Islet-1 was dramatically reduced in cardiac progenitor cells when transfected with Islet-1 RNA interference vector to inhibit Islet-1 (A). The mRNA levels for Mef2c and Tbx5 were significantly reduced in the cells when Islet-1 expression was inhibited with RNAi as compared with the controls. There was no significant difference in GATA4 mRNA level in the cells with Islet-1 knockdown with RNAi (B). *p < 0.05, n=3-5.

To evaluate the role of Islet-1 in the expression of cardiac development-related transcription factors in cardiac progenitor cells, the cells were transfected with a lentivrius with Islet-1 RNA interference vector to inhibit Islet-1. As shown in Figure **3A**, the transfection efficiency was 47.3% as evaluated with flow-cytometry. PCR analysis showed that the Islet-1 mRNA level was dramatically reduced in the cells transfected with Islet-1 RNAi vector by 84.5% (p<0.05) (Figure **4A**), indicating that the transcriptional expression of Islet-1 was effectively inhibited with the vector. No significant change in the Islet-1 mRNA level was observed in the cells transfected with control vector (p>0.05). No significant difference in the morphology of the cells was identified after lentivrius transfection (Figure **3B**), suggesting that neither the transfection nor lentivirus had significant toxic effect on the cells. 

Quantitative RT-PCR analysis showed that the mRNA levels of the cardiac development-related transcription factors, Mef2c (0.29±0.03) and Tbx5 (0.02±0.00) were significantly reduced in the cells transfected with Islet-1 RNAi vector as shown in Figure **4B** (*p*<0.05). There was no significant change in the expression profiles of these transcription factors in the control cells (*p*>0.05). However, the mRNA level of GATA4 was not significantly changed in the cells with Islet-1 inhibition (*p*>0.05).

### Inhibition of Islet-1 selectively decreased histone H3 acetylation level in Mef2c promoter region in cardiac progenitor cells

**Figure 5:**
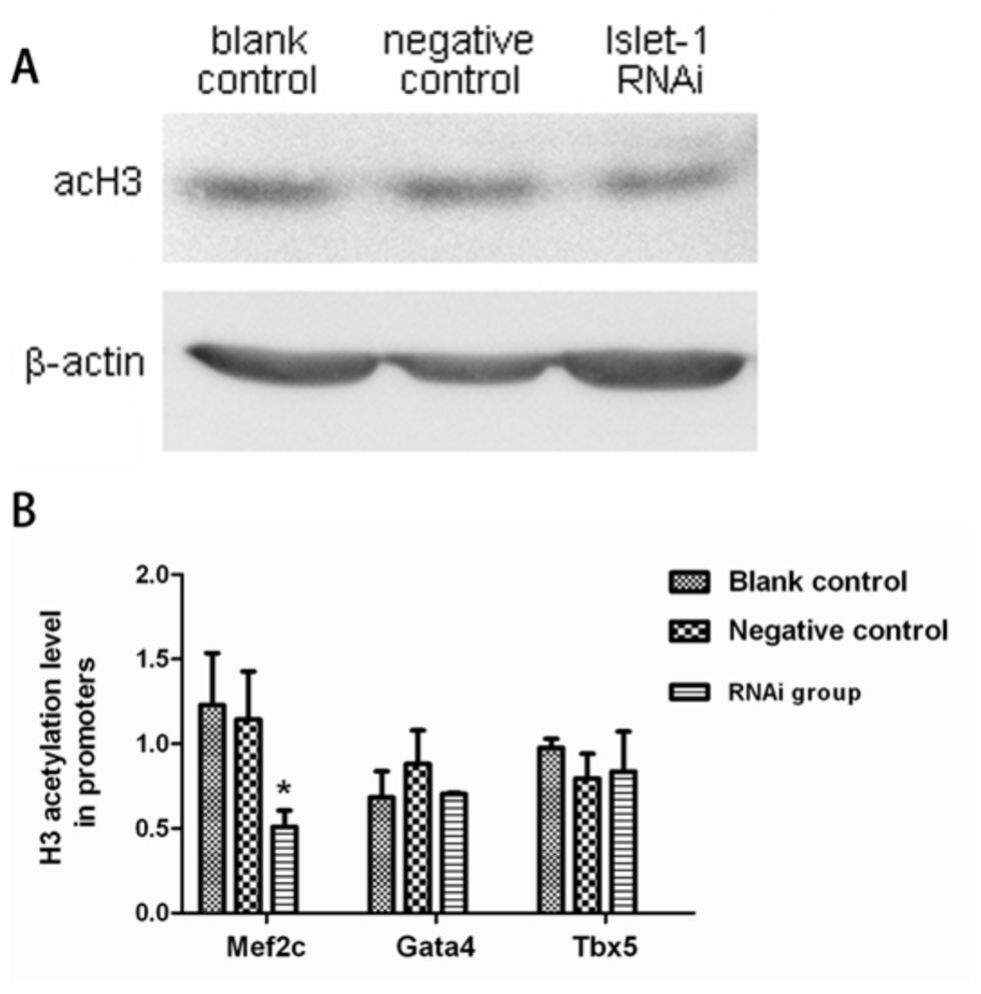
Acetylation level of histone H3 in cardiac progenitor cells after inhibiting Islet-1 expression. Western blotting analysis showed that detectable level of histone H3 acetylation was present in cardiac progenitor cells, and was not significantly changed when Islet-1 expression was inhibited (A, *p*>0.05, n=3). However, the DNA quantity of Mef2c in the promoter region immunoprecipitated with acH3 antibody was significantly decreased in the cells transfected with Islet-1 RNAi as compared with the controls, while no difference was observed in the DNA quantity of GATA4 and Tbx5 in the cells (B). *p<0.05, n=3.

Western blotting analysis showed that acetylated histone H3 was present in cultured cardiac progenitor cells as expected (Figure **5A**). The total acetylation level of histone H3 was not significantly changed in the cells transfected with Islet-1 RNAi vector (*p* > 0.05), suggesting that Islet-1 inhibition had no impact on the total amounts of acetylated histone H3 in the whole chromatin. 

We then investigated whether the level of acetylated histone H3 was changed in the specific promoter regions of cardiac development-related transcription factors in the cells when Islet-1 was inhibited. Using ChIP and quantitative RT-PCR technique, we found that acetylated histone H3 level (0.51±0.10) was significantly decreased in the promoter region of Mef2c in the cells transfected with Islet-1 RNAi vector by 55.3% over control (1.14±0.28) (*p*<0.05) (Figure **5B**). There was no significant change in acetylated histone H3 level in the control cells (*p*>0.05), indicating that the reduced level of acetylated histone H3 was due to inhibition of Islet-1. However, there was no changes in the acetylated histone H3 level in the promoter regions for GATA4 or Tbx5 in the cells when Islet-1 was inhibited (*p*>0.05). 

### Inhibition of Islet-1 selectively decreased the Mef2c DNA quantity in the complex with p300 in cardiac progenitor cells

**Figure 6:**
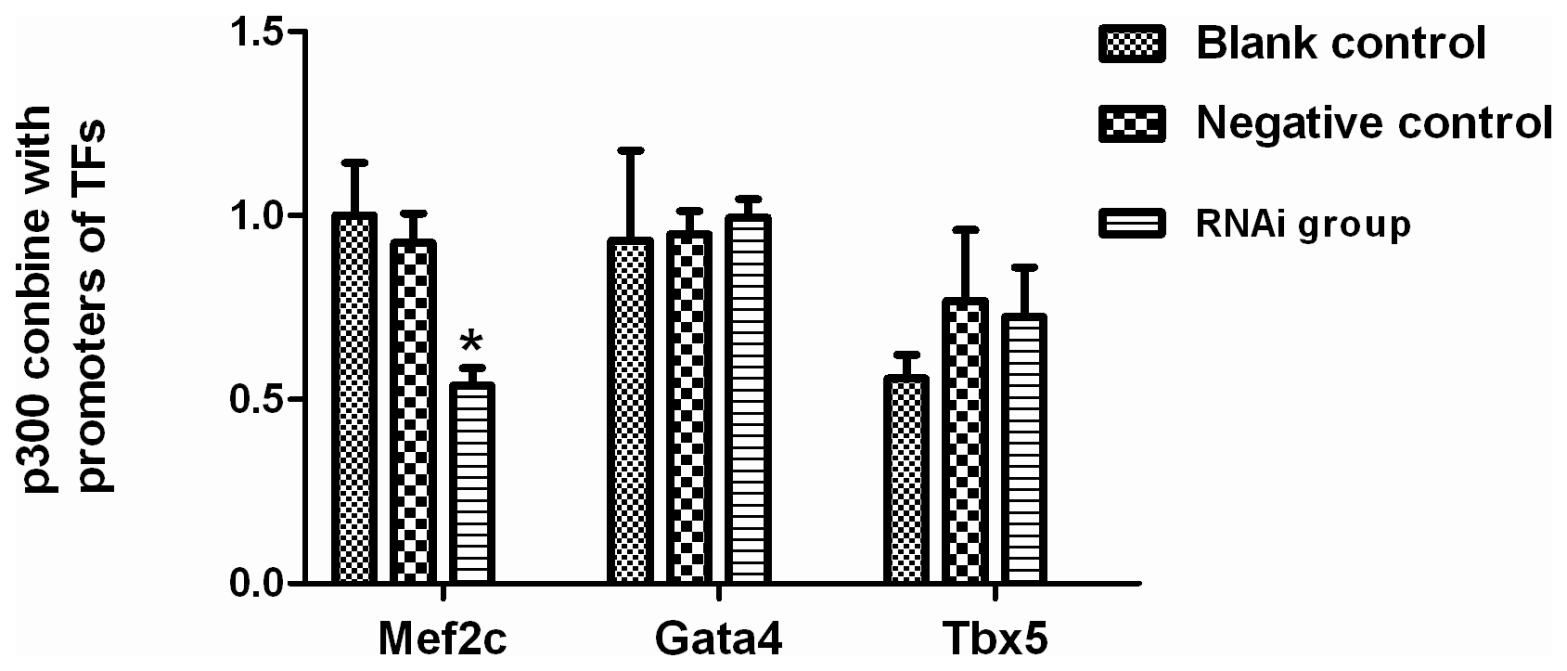
Interaction of p300 with the promoter regions of Mef2c, GATA4 and Tbx5 in cardiac progenitor cells. The DNA quantity of Mef2c in the promoter regions immunoprecipitated using p300 antibody was significantly decreased in the cells transfected with Islet-1 RNAi as compared with the controls. However, no difference in the DNA quantity of GATA4 and Tbx5 was observed in the cells with or without transfection with Islet-1 RNAi. * *p* < 0.05, n=3.

To evaluate the role of Islet-1 in the interaction of p300 with promoter regions of cardiac development-related transcription factors in cardiac progenitor cells, Islet-1 was inhibited with specific Islet-1 RNAi. The DNA quantity for each transcription factor was analyzed with quantitative PCR in the complex after precipitation with p300 antibody. As shown in Figure **6**, the DNA quantity of Mef2c (0.54±0.05) was significantly decreased in the cells when Islet-1 was inhibited by almost 50% over control (*p*<0.05). However, there was no significant change in the DNA level for GATA4 or Tbx5 in the cells transfected with Islet-1 RNAi vector (*p*>0.05). No significant difference in DNA levels for Mef2c, GATA4 or Tbx5 was identified in the cells transfected with the control vector (*p*>0.05). These data suggested that inhibition of Islet-1 selectively reduced the binding of p300 to the promoter region of Mef2c in cultured cardiac progenitor cells. 

## Discussion

LIM-Homeodomain transcription factor Islet-1 was first reported as an insulin gene enhancer protein [19]. Heart is one of the first functional organs developed during embryogenesis. Islet-1 is mainly expressed in the early cardiac progenitor cells, and plays a critical role in cardiac development [20-24]. Expression of Islet-1 is down-regulated in most cardiac progenitors when they differentiate, and is not detectable in differentiated cardiomyocyte [11,14]. Similar results on Islet-1 expression profile were observed in the present study. Interestingly, transfection efficiency of Islet-1 RNAi was 47.3%, but mRNA level of Iselet-1 was significantly reduced in cells transfected with Islet-1 RNAi vector by 84.5%. However, this was not paradoxical, because actually we inspected GFP expression by both eye inspection under microscope and FCM. The transfection efficacy of our lentivirus is very high (more than 80%) in cardiac progenitor cells based on our eye examination. However due to that no cell sorting was done so the FCM data did not exclude dead cells or other particles that display no GFP signal in FCM assays resulting in a lower positive GFP ratio. 

Heart formation is an extremely complex process which is regulated by specific cardiac development-related transcription factors including Mef2, GATA4, and Tbx5. Islet-1 is important as a transcription factor in early embryonic heart development with extensive interactions with cardiac development-related transcription factors[25]. It is reported that Mef2c is a direct transcriptional target of Islet-1 during mouse embryonic development [26]. In the present study, we observed that the expression of Mef2c was reduced as a result of inhibition of Islet-1 expression, which was consistent with previous observation[26]. We also found that the expression of Tbx5 was reduced in cardiac progenitor cells when Islet-1 was inhibited with specific RNAi, suggesting that Islet-1 might regulate the expression of Tbx5 as well. This was the first time to demonstrate that Islet-1 was involved in the regulation of Tbx5 expression. It is reported that Tbx5 is mainly involved in the development of the first heart field [27]. Although Islet-1 plays an important role in cardiac development of the second heart field[11,28], it is also reported to contribute to the development of some specific regions in the left ventricle from the first heart field[14]. Further studies are needed to further establish the exact role of Islet-1 in Tbx5 expression. Interestingly, GATA4 expression was not affected when Islet-1 was inhibited, indicating that Islet-1 selectively participated in the regulation on the expression of Mef2c and Tbx5, not GATA4, in cardiac progenitor cells. 

Histone H3 acetylation in the promoter region is a dynamic process [9]. Modification of histone acetylation is an important mechanism for the regulation of gene expression. The DNA templates on chromatin structure are more accessible to transcriptional factors to promote the gene expression when acetylation of amino-terminal lysine residues of histone occurs. In contract, histone deacetylation leads to a compact structure of chromatin, which suppresses or silences gene expression [29,30]. Modification of histone acetylation is a dynamic process that is regulated by HATs and HDACs. HATs p300, a subtype of HATs, is expressed dynamically in the developing hearts with the expression pattern similar to that for Mef2c, GATA4 and Tbx5 [1,8]. Inhibition of p300 activity leads to decreased histone acetylation and attenuates the expression of cardiac development-related transcription factors [9]. HATs p300 (-/-) or p300(+/-) mice are lethal at their embryonic or neonatal stages with impaired formation of cardiovascular system, lungs and small intestine [31]. These data suggest that p300 plays an important role in the development of heart and other organ systems. Modification of histone acetylation also interferes with the development of brain and liver [32,33], indicating that the effects of histone acetylation are not specific for cardiovascular development. How histone acetylation by p300 specifically regulates the expression of cardiac development-related factors at the key time point during heart development remains unclear. 

Islet-1 is an important transcription factor for cardiac development through mediating extensive interactions between DNA and proteins [16]. In the present study, we observed that Islet-1 peak expression occurred on day E14.5 in mouse embryonic heart, was present in the promoter regions for cardiac development-related factors Mef2c, GATA4 and Tbx5 precipitated with p300 antibody. When Islet-1 was inhibited with specific RNAi in cardiac progenitor cells, the expression of cardiac development-related transcription factors Mef2c and Tbx5, but not GATA4, was significantly suppressed in these cells along with selective reduction in the acetylation level of histone H3 in the Mef2c promoter region. These data suggested that Islet-1 was selectively involved in the regulation of Mef2c and Tbx5 expression independent of the overall level of histone H3 acetylation in cardiac progenitor cells. However, the acetylation level of histone H3 in the promoter region of Mef2c, not GATA4 and Tbx5, was markedly reduced when Islet-1 was inhibited, indicating that Islet-1 might regulate histone acetylation in the promoter of Mef2c, but not GATA4 and Tbx5. The data from present study also showed that the level of Mef2c DNA, not GATA4 and Tbx5, in the complex associated with p300 was significantly decreased in the cells with Islet-1 knockdown. Taken together, it appeared to be that inhibition of Islet-1 selectively attenuated the interaction of p300 with Mef2c promoter, then, suppressed histone H3 acetylation in the promoter of Mef2c, leading to down-regulation of Mef2c expression. Therefore, Islet-1 seemed to be an assistant factor that mediated the modification of histone acetylation and regulation of Mef2c expression via assisting p300 on specifically targeting the promoter of Mef2c. However, further research is needed to confirm this mechanism. Inhibition of Islet-1 also suppressed the expression of Tbx5 in cardiac progenitor cells. Clearly, histone H3 acetylation was not involved in the action of Islet-1 on Tbx5 since there was no change in histone H3 acetylation level when Islet-1 was inhibited in the cells. Further studies are needed to investigate the underlying mechanisms. 

## Conclusion

In the present study, we demonstrated that Islet-1 was dynamically expressed and associated with p300 in mouse embryonic heart. Inhibition of Islet-1 selectively suppressed the expression of Mef2c in association with selective inhibition of histone H3 acetylation in Mef2c promoter region and decreased p300 binding to the promoter region in cardiac progenitor cells. These data suggested that Islet-1 might function as an assistant factor that was involved in the regulation of histone acetylation and Mef2c expression via assisting p300 on interacting specifically with the promoter of Mef2c. 
